# Liver Injury Due to Hormonal Contraceptives in the Modern Era

**DOI:** 10.1111/liv.70945

**Published:** 2026-11-02

**Authors:** Raj Vuppalanchi, A. Sidney Barritt, Anjali Rai, Huiman Barnhardt, Sherry Gu, Yi‐Ju Li, Naga Chalasani, Drug‐Induced Liver Injury Network, Naga Chalasani, Marwan Ghabril, Raj Vuppalanchi, Bob Fontana, Alisa Likhitsup, Vincent Chen, Victoria Shakhin, Paul Watkins, Sid Barritt, B. B. Behera, Jacquee Simpson, Herb Bonkovsky, Dee Faust, Andrew Stolz, Lily Dara, Neil Kaplowitz, Susan Milstein, Gina Choi, Jacqueline Nguyen, Angela Yeh, Dina Halegoua‐DeMarzio, Victor Navarro, Manish Thapar, Manisha Verma, Katrin Koy‐Pan, Verma Manisha, Cynthia Miller, Angela Pallotto, Christopher Koh, Beverly Niles, Jaha Norman, Huiman Barnhart, Matthew Baum, Jiezhun Gu, Yi‐Ju Li, Michelle McClanahan‐Crowder, Jeremy Mercer, Patricia Montana, Viviana Moreno, Eilene Pham, Hoss Rostami, Sheri Ussery, Kaye Williams, Stephanie Wu, Peter Hoffmann, Kidney Diseases, José Serrano, Jay Hoofnagle, Averell H. Sherker

**Affiliations:** ^1^ Division of Gastroenterology and Hepatology Indiana University School of Medicine Indianapolis USA; ^2^ UNC Health Chapel Hill USA; ^3^ NIH/NIDDK Bethesda USA; ^4^ Duke Clinical Research Institute Durham USA

**Keywords:** birth control, drug‐induced liver injury, hepatotoxicity, oral contraceptive

## Abstract

**Background and Aims:**

Hormonal agents and their association with liver injury remain a concern in clinical practice. This study aimed to characterise the biochemical and clinical features of hormonal agent‐associated liver injury and compare them with a control group of age‐ and gender‐matched cases of liver injury caused by non‐hormonal drugs.

**Methods:**

Of 2123 adjudicated drug‐induced liver injury cases enrolled in the Drug‐Induced Liver Injury Network (2004–2024), 20 high‐confidence cases (1.3%) were attributed to hormonal agents. These cases were compared with age‐ and sex‐matched non‐hormonal drug‐induced liver injury (DILI) controls, and analyses assessed clinical features, biochemical patterns, outcomes, HLA alleles and candidate genetic variants associated with cholestatic liver disease.

**Results:**

The median age of women was 22 years. Most presented within 2–12 weeks with jaundice and pruritus, predominantly with cholestatic or mixed patterns of liver enzyme elevations, but a hepatocellular pattern was not uncommon at 28%. The route of administration did not appear to influence DILI risk. Half underwent a liver biopsy, and half required hospitalization, but none died or required liver transplantation. Compared with other DILI cases, hormonal agent‐associated liver injury was more often cholestatic, with jaundice and pruritus, and less frequently associated with fever or rash. No HLA association was identified, although the intronic *MROH2A* variant rs6722064 ranked as the top potential candidate for increased risk [OR (95% CI) = 3.58 (1.67, 7.65), *p* = 0.001].

**Conclusions:**

Hormonal agent‐associated liver injury presents with a distinctive mixed or cholestatic pattern and often lacks the inflammatory features seen in other DILI cases. These findings underscore the need for greater clinical awareness to improve patient outcomes.

## Key Points/Highlights

- Hormonal agent‐associated liver injury was rare, accounting for 1.3% of high‐confidence DILI cases in the current cohort.
- The clinical phenotype was predominantly cholestatic or mixed, although a hepatocellular pattern was observed in 28% of cases.
- Liver injury typically developed within weeks of exposure, with a median latency of approximately 5 weeks.
- Liver injury occurred across diverse hormonal formulations and routes of administration, including oral, transdermal and subdermal contraceptives.
- No significant human leukocyte antigen (HLA) associations were identified; however, candidate non‐HLA genetic variants implicated in bile acid homeostasis and cholestatic pathways were observed.

## Lay Summary

This study reports that liver injury can occur with oestrogen‐only, progesterone‐only or combination hormonal agents/contraceptives, including patches and implants. Most affected individuals developed jaundice (yellowing of the skin or eyes) and itching within 3 months of starting the hormonal medication. None died or required a liver transplant.

## 1 Introduction

Early formulations with higher doses of oestrogen were commonly associated with liver enzyme elevations [1]. These cases of drug‐induced liver injury (DILI) presented with cholestatic liver injury characterised by jaundice and severe pruritus, often developing within 1–3 months of starting treatment [2, 3, 4]. Liver biopsy findings usually showed bland cholestasis, and symptoms usually resolved after stopping the medication. The Swedish population‐based study analysed national adverse drug reaction data from 1966 to 1990 across millions of oral contraceptive treatment cycles. It showed a marked decline in reported liver reactions as high‐dose oestrogen formulations were replaced by lower‐dose preparations [5]. Although newer OCs with lower oestrogen doses have been shown in clinical trials and drug safety registries to reduce the risk of liver enzyme elevations, concerns about hepatic safety remain due to isolated case reports or small case series that lack thorough exclusion of other causes and comprehensive follow‐up [3, 4, 5]. A recent multicentre study from India reported that 19 of the 43 cases of liver injury caused by hormonal agents involved combination oral contraceptives (OCs) [6]. The study observed a latency period of 2–3 months, with injury resolution approximately 2 months after discontinuation of the implicated medication [6]. Interestingly, all three patterns of liver enzyme elevation were observed [6]. Additionally, two deaths were reported with progesterone‐only preparations (medroxyprogesterone 10 mg and megestrol acetate 160 mg), with no deaths linked to combination OCs [6, 7].

The establishment of a multicentre DILI registry, such as the Drug‐Induced Liver Injury Network (DILIN) in the U.S., has significantly enhanced the ability to identify rare cases of idiosyncratic DILI through standardised protocols and prospective follow‐up [8, 9, 10]. In this analysis, we examined cases of DILI where hormonal agents were used for oral contraception or dysfunctional uterine bleeding. Our aim was to characterise the hormonal agents implicated in liver injury, describe their clinical, biochemical, and histologic (when available) features and outcomes, compare them with matched controls, and evaluate associations with HLA alleles and candidate genes linked to cholestatic liver disorders.

## 2 Methods and Materials

The DILIN, described in prior publications, is a multicentre network of 5 to 8 U.S. medical centres funded by the NIH to identify and enrol suspected cases of idiosyncratic DILI [8, 9, 10]. Institutional Review Boards approved the DILIN Prospective Study (NCT00345930) at all participating centres [8, 9, 10]. The study inclusion and exclusion criteria, enrolment, and causality adjudication process have been previously reported [8, 9, 10]. The role of hormonal contraceptives, like other implicated agents, in causing liver injury was evaluated through detailed case narratives and reporting forms [8, 9, 10]. The likelihood of causation was quantified using scores from 1 (definite) to 5 (unlikely) by 3 independent investigators, who reached consensus if there was any discordance [8, 9, 10]. This study selected cases with high confidence in DILI (causality scores 1–3) to examine demographic, clinical, biochemical, and histologic characteristics and to identify signature patterns. Cases of chronic liver injury were identified based on DILIN criteria (i.e., evidence of persistent laboratory, radiological, or pathological abnormalities 6 months after DILI onset) [8, 11]. DILI cases of age‐ (±10 years) and body mass index‐ (±5 kg/m^2^) matched women from the DILIN database, who were not treated with hormonal agents or herbal/dietary supplements and had high‐confidence adjudication scores, were selected as controls for comparison (Table 1). Details of genome‐wide single‐nucleotide polymorphism (SNP) array analysis and HLA sequencing have been reported [12, 13, 14]. Specifically, HLA sequencing of DNA extracted from whole blood samples was performed on the Illumina MiSeq platform at the Vanderbilt Immunogenomics, Microbial Genetics, and Single Cell Technologies core (IMGSCT).

**TABLE 1:** Select demographics and clinical features of cases of drug‐induced liver injury (DILI) in the hormonal contraceptive‐induced liver injury group and age‐ and gender‐matched group consisting of DILI cases from non‐hormonal agents.

Feature	Hormonal agent (*N* = 20)	Control (*N* = 60)	*p*
Age (years), median (Q1, Q3)	22.1 (18.3, 29.4)	22.1 (18.3, 29.4)	0.96
Female sex	100%	100%	NC
Race			
White	15/20 (75%)	48/60 (80%)	0.384
Black	3/20 (15%)	6/60 (10%)	
Asian	0/20	4/60 (6.7%)	
Other	2/20	3/60 (3.3%)	
Hispanic	2/20 (10%)	6/60 (10%)	> 0.999
BMI (kg/m^2^)	26.8 (7.8)	25.1 (6.4)	0.78
History of drug allergies	4/20 (20%)	18/60 (30%)	0.39
Alcohol use (any)	5/20 (25%)	25/56 (44.6%)	0.123
Drug start to the onset of DILI (days)			
Median (Q1, Q3)	36 (26, 72)	60 (27, 163)	0.334
Mean (SD)	107 (169)	177 (310)	
≤ 1 week	0/20	3/60 (5.0%)	0.532
2–4 weeks	6/20 (30.0%)	13/60 (21.7%)	
5–12 weeks	10/20 (50.0%)	21/60 (35.0%)	
13–24 weeks	1/20 (5.0%)	8/60 (13.3%)	
> 24 weeks	3/20 (15.0%)	15/60 (25.0%)	
Causality score			
Definite	5/20 (25.0%)	18/60 (30.0%)	0.912
Highly likely	11/20 (55/0%)	31/60 (51.7%)	
Probable	4/20 (20.0%)	11/60 (18.3%)	
Symptoms			
Jaundice	16/20 (80%)	29/60 (48%)	0.013
Nausea	8/20 (40%)	35/60 (58%)	0.154
Fever	0/20	21/60 (35%)	0.002
Abdominal pain	5/20 (25%)	35/60 (58%)	0.010
Rash	3/20 (15%)	19/60 (32%)	0.148
Itching	16/20 (80%)	23/60 (38%)	0.001

### 2.1 Statistical Analysis

Descriptive statistics for demographics and clinical outcomes were summarised for cases and controls as frequencies (%) for categorical variables and means with standard deviations (SDs) or medians with ranges for continuous variables. Chi‐square and two‐sample *t*‐tests were performed to compare variable differences between the two groups. HLA alleles with very low allele frequency (AF) in cases or controls were excluded. Fisher's exact tests were performed to assess the association between HLA alleles and DILI caused by hormonal agents. For identifying genetic risk variants for hormonal‐agent DILI, we targeted a list of known cholestasis‐related genes. A total of 82 genes were assessed (Table S1). Variants located within each gene region, including a ±10 kb boundary, were analysed. Similarly, Fisher's exact tests were performed to test AF differences between cases and controls. *Q*‐value, a method for controlling the false discovery rate, was computed to adjust for multiple testing. HLA and genetic variants meeting *Q*‐value < 0.15 were considered significant. Given the small number of cases and the lack of independent validation, we consider HLA alleles with *p* < 0.05 and SNPs with *p* < 0.01 as suggestive risk variants for DILI associated with hormonal agents.

## 3 Results

Between September 2004 and October 2024, the DILIN prospective study enrolled 2123 suspected cases of DILI, with 6 months of follow‐up and formal causality adjudication. Initially, 34 cases of hormonal agent‐associated liver injury were identified. However, 14 cases were excluded from the current analysis for the following reasons: 4 involved tamoxifen, a selective oestrogen receptor modulator; 1 involved mifepristone, used to treat adrenal hypercortisolism in a male patient; and 9 had possible or unlikely causality scores. The remaining 20 cases made up 1.3% of the 1553 cases scored as probable, highly likely, or definite DILI (high‐confidence cases). These 20 cases involved agents such as oestrogen only (3/20, 15%), natural progesterone only (1/20, 5%), and combinations like ethinyl oestradiol with different types of progestins, including norethindrone (*n* = 3), norgestrel (*n* = 1), levonorgestrel (*n* = 3), norelgestromin (*n* = 1), norethisterone acetate (*n* = 1), drospirenone (*n* = 3), medroxyprogesterone with norethindrone (*n* = 1), or norgestimate (*n* = 3). One individual received the hormonal agent via a topical patch, while another used a subdermal implant. These cases were enrolled steadily from 2005 to 2021, with no more than three cases enrolled annually.

This group of 20 patients with liver injury from hormonal agents was entirely female, with a median age of 22 years. Most were White (75%), followed by Black (15%) and Hispanic (10%). The majority developed DILI within 2–12 weeks of starting the drug (80%), with a median latency of 36 days (IQR 26.0, 71.5) (Table 1). These patients were symptomatic with symptoms of cholestasis, presenting with jaundice (80%) and itching (80%), often accompanied by nausea (40%) and abdominal pain (25%). Liver enzyme patterns as defined by *R* values were mainly cholestatic (39%) or mixed (33%), with initial mean ALT levels of 353 U/L, AST 187 U/L, alkaline phosphatase 213 U/L, and bilirubin 7.5 mg/dL. The *R* values averaged 5 and ranged from 0.6 to 22.8, being ≤ 2.0 (cholestatic) in 39%, 2.1–4.9 in 33% (mixed), and ≥ 5.0 in 28% (hepatocellular) (Table 2). Autoimmune markers were positive in 24% of cases. Liver biopsies were performed in 50% of the cohort. Severity assessments showed that 50% were moderately severe and hospitalized, while 5% experienced liver‐related decompensation; however, there were no fatalities or transplants. Chronic DILI was seen in 2 patients and corticosteroids were used in 3.

**TABLE 2:** Biochemical features of cases of drug‐induced liver injury (DILI) in the hormonal agent‐induced liver injury group and age‐ and gender‐matched group consisting of DILI cases from non‐hormonal agents. Laboratory values are given as means with standard deviations.

Feature	Hormonal agent (*N* = 20)	Control (*N* = 60)	*p*
Initial ALT (U/L)	353 (337)	952 (969)	0.001
Initial AST (U/L)	187 (183)	772 (846)	< 0.001
Initial ALP (U/L)	213 (157)	196 (160)	0.324
Initial total bilirubin (mg/dL)	7.5 (5.5)	3.9 (4.4)	0.003
Initial *R* value	5.0 (5.8)	16.0 (20.4)	< 0.001
Enzyme pattern			0.003
Hepatocellular (*R* ≥ 5)	5/18 (28%)	40/57 (70%)	
Mixed (*R* > 2 to < 5)	6/18 (33%)	11/57 (19%)	
Cholestatic (*R* ≤ 2)	7/18 (39%)	6/57 (11%)	
Peak ALT (U/L)	504 (397)	1311 (1379)	< 0.001
Peak AST (U/L)	326 (458)	1308 (1894)	< 0.001
Peak ALP (U/L)	280 (176)	269 (229)	0.386
Peak total bilirubin (mg/dL)	13.0 (9.2)	8.4 (9.8)	
Peak INR	1.2 (0.5)	1.6 (1.6)	
Peak eosinophils (cells/μL)	203.1 (216.4)	401.7 (794.4)	0.193
Peak eosinophils > 500 cells/μL	1/20 (5%)	10/57 (18%)	0.271
ANA	2/20 (10%)	28/58 (48%)	0.002
SMA	4/17 (24%)	12/56 (21%)	0.013
Severity assessment			
Mild (anicteric)	4/20 (20%)	23/60 (38%)	0.088
Moderate (jaundiced)	5/20 (25%)	8/60 (13%)	
Moderate and hospitalized	10/20 (50%)	15/60 (25%)	
Severe (decompensation)	1/20 (5%)	11/60 (18%)	
Fatal (death or transplant)	0/20	3/60 (5%)	
Liver biopsy	10/20 (50%)	26/60 (43%)	0.604
Hospitalization	9/20 (45%)	41/60 (68%)	0.062
Treated with prednisone or corticosteroids	3/20 (15%)	21/60 (35%)	0.091
Chronic DILI	2/19 (11%)	8/52 (15%)	0.719
Death or liver transplant	0	0	

Abbreviations: ALP, alkaline phosphatase; ALT, alanine aminotransferase; ANA, antinuclear antibody; AST, aspartate aminotransferase; DILI, drug‐induced liver injury; INR, international normalized ratio; *R* value, ratio used to classify DILI pattern: [ALT/ULN] ÷ [ALP/ULN]; SMA, smooth muscle antibody.

In the hormonal agent group, notable differences were observed compared with the control group of other causes of DILI, particularly in clinical presentation and symptom profile. Patients in the hormonal agent group exhibited a higher prevalence of jaundice (80% vs. 48%, *p* = 0.01) and itching (80% vs. 38%, *p* = 0.001). In contrast, fever was reported only in the control group (0% vs. 35%, *p* < 0.01). Abdominal pain was also less common in the hormonal agent group compared to controls (25% vs. 58%, *p* = 0.01). Factors such as race, history of drug allergies, and alcohol use did not differ significantly between the groups. The onset of DILI occurred sooner in the hormonal agent group, with a median of 6 days versus 60 days in the control group, although this difference was not statistically significant (*p* = 0.30). Several important differences in liver‐related parameters were noted. In the hormonal agent group, liver injury was mainly cholestatic or mixed enzyme pattern, with a significantly higher proportion of cholestatic injury than the control group (39% vs. 11%, *p* < 0.01). Initial total bilirubin levels were also significantly higher in the hormonal agent group (7.5 vs. 3.9 mg/dL; *p* < 0.01), indicating more severe cholestasis. The initial ALT and AST levels were lower in the hormonal group compared to the control group (353 vs. 952 U/L, *p* = 0.001; 186 vs. 772 U/L, *p* < 0.001). Autoimmune markers were less common in the hormonal agent group, with significantly lower ANA positivity (10% vs. 48%, *p* < 0.01). Illustrative cases from each category (combined OCs, progesterone alone, topical patch and subdermal implant) are summarised in Table 3.

**TABLE 3:** Illustrative cases of liver injury associated with hormonal contraceptive agents, stratified by agent and route of delivery.

Case/suspect agent	Demographics	Latency to onset	R value/pattern	Peak biochemistry	Clinical findings	Liver histology	Outcome
Case 1 Progesterone‐only OCP (norethisterone)	25‐year‐old woman White BMI 29.6 kg/m^2^	~1 month to symptom onset Total exposure ~60 days	*R* = 2.3 (mixed pattern)	AST 172 U/L ALT 525 U/L ALP 387 U/L T. bilirubin 1.3 mg/dL	Abdominal pain, jaundice Cholecystectomy (neg. cholangiogram) MRCP: no biliary abnormality Viral hepatitis serologies negative	Not performed	Improved but not normalized by day 57 (AST 127 U/L, ALT 296 U/L, ALP 238 U/L, T. bili 2.2 mg/dL) Persistent pruritus; LSM 7.2 kPa Lost to follow‐up
Case 2 Intravenous/oral oestrogen (Premarin → estradiol)	18‐year‐old woman Adopted (family history unknown)	Days after IV oestrogen; oral therapy continued weeks Chronic course over years	Not reported (cholestatic course)	T. bilirubin 3.6 → 13.0 mg/dL AST 115 → 215 U/L ALT 129 → 223 U/L ALP 201 → 416 U/L ANA 1:80 (+)	Uterine bleeding post‐IUD removal Jaundice; MRCP CBD stone (not confirmed by EUS) HVPG 8 mmHg; no varices Recurrent jaundice with pregnancy ~4 years later	Vanishing bile duct syndrome Chronic cholestasis, Stage 2 fibrosis Preserved bile ducts	Removed from transplant list after initial improvement Persistent hyperbilirubinaemia (~11 mg/dL) Now being re‐evaluated for liver transplant
Case 3 Etonogestrel implant + short‐course COC	20‐year‐old woman White History of intrahepatic cholestasis of pregnancy	Soon after implant placement +2‐week COC course	Not reported (cholestatic pattern)	T. bilirubin peak 14.9 mg/dL (bland cholestasis)	Pruritus recurrence Cholecystectomy for gallstones (no improvement) ERCP normal Sporadic green tea extract use	Bland cholestasis No necrosis, granulomas, or fibrosis ABCB11 c.2610 + 5G>A variant (novel)	Full normalization of liver tests
Case 4 Transdermal contraceptive patch	29‐year‐old woman Hispanic History of jaundice in 3 prior pregnancies	~1 month after patch initiation	Not reported (minimally elevated ALT/AST/ALP — cholestatic pattern)	T. bilirubin 8.4 mg/dL ALT, AST, ALP minimally elevated	Fatigue, dysuria, painless jaundice, severe pruritus No biliary dilation on imaging Viral/autoimmune serologies negative No concurrent drugs/supplements	Cholestasis with minimal chronic portal inflammation No fibrosis	Pruritus resolved with rifampin Liver tests normalized

*Note: R* value = (ALT/ULN) ÷ (ALP/ULN), used to classify DILI as hepatocellular (≥ 5), mixed (2–5) or cholestatic (≤ 2).

Abbreviations: COC, combined oral contraceptive; ERCP, endoscopic retrograde cholangiopancreatography; EUS, endoscopic ultrasound; HVPG, hepatic venous pressure gradient; LSM, liver stiffness measurement; MRCP, magnetic resonance cholangiopancreatography; OCP, oral contraceptive pill; ULN, upper limit of normal.

Of the 15 cases with dosages identified, low‐dose monophasic formulations accounted for the majority (*n* = 8) and demonstrated the widest range of *R*‐values (0.5–36.6), encompassing cholestatic, mixed, and hepatocellular patterns, including the single highest *R*‐value observed in the cohort (Figure 1). Low‐dose triphasic cases (*n* = 3) were predominantly cholestatic or mixed (*R* = 1.8–2.7), with one hepatocellular outlier (*R* = 22.8). The single high‐dose monophasic case was cholestatic (*R* = 1.4), while the two ultra‐low‐dose monophasic cases spanned both cholestatic and hepatocellular patterns (*R* = 0.5 and 6.0, respectively).

**FIGURE 1:**
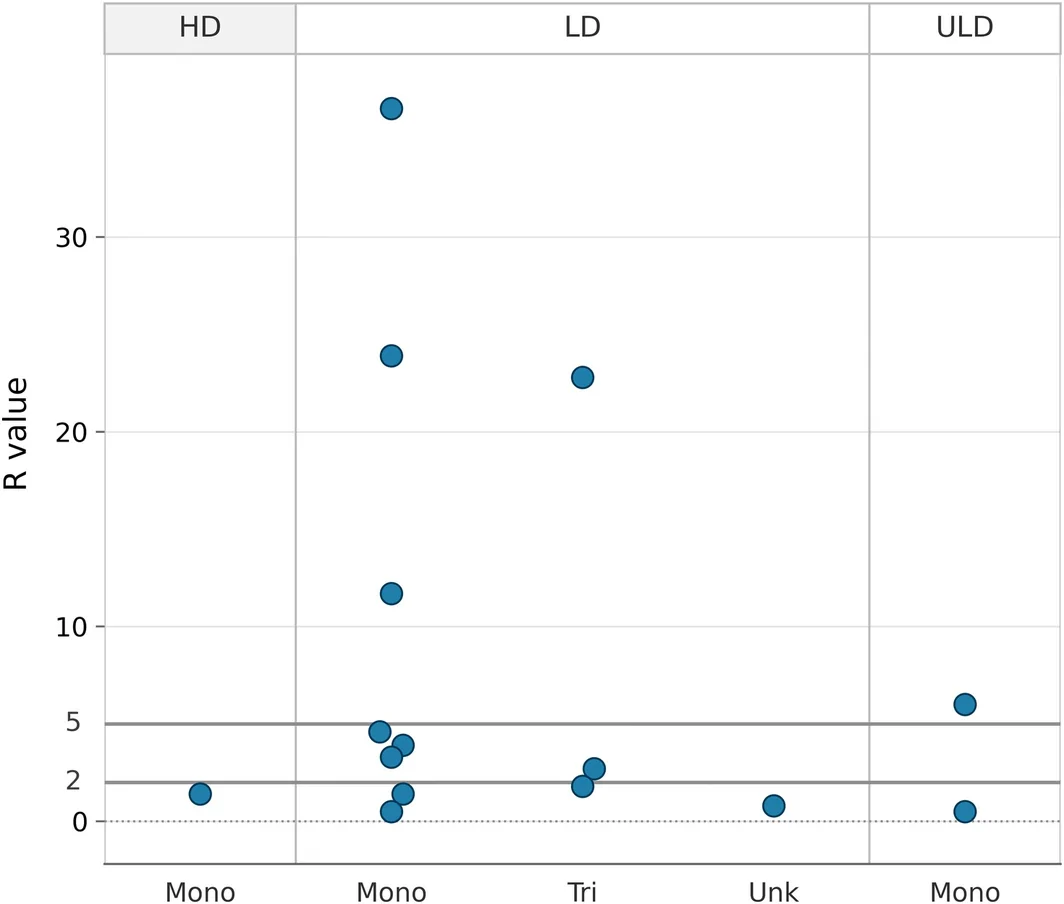
The figure illustrates the relationship between the oestrogen dosage in the hormonal agents, the type of action and the initial *R*‐value. The different dosage levels [high dose (HD), low dose (LD), ultra‐low dose (ULD)], contraceptive mode of action in phases (monophasic, triphasic, unknown) and the initial *R* values are represented in the figure. Each dot represents a patient. This visualization provides insights into how initial *R* values vary across dosage and type combinations.

### 3.1 HLA and Candidate Gene Associations

In the GWAS data, we did not find any HLA or genetic variants that met the significance threshold (*Q*‐value < 0.15). Additionally, no HLA alleles met the *p* < 0.05 threshold for potential risk alleles. However, candidate gene association analysis identified six genes with variants showing *p* < 0.01 as potential risk factors for DILI caused by hormonal agents; the top variant within each gene, with the smallest *p*‐value, is listed in Table 4. Among these, SNP rs6722064 in the *MROH2A* gene ranked highest [OR (95% CI) = 3.58 (1.67, 7.65), *p* = 0.001]. The minor allele of the top variants in *MROH2A, NR1H4, NPHP1* and *TJP2* was associated with increased risk of DILI (OR > 1), while rs3795860 in *ABCG8* [OR (95% CI) = 0.31 (0.14, 0.71), *p* = 0.005] and rs2035648 in *AKR1D1* [OR (95% CI) = 0.28 (0.11, 0.68), *p* = 0.004] showed a protective effect, reducing the risk of DILI.

**TABLE 4:** The top genetic variants reaching *p*‐value ≤ 0.005, demonstrating potential association with hormonal agent‐induced DILI from the candidate gene association analysis.

Chr:BP	SNP	Gene	Location	Allele	Case AF	Control AF	OR (95% CI)	*p*
2:233780897	rs6722064	*MROH2A*	Intronic	G	0.55	0.25	3.58 (1.67, 7.65)	0.001
7:138115259	rs34315519	*AKR1D1*	Intronic	A	0.18	0.44	0.28 (0.11, 0.68)	0.004
2:43871716	rs3795860	*ABCG8*	Intronic	C	0.23	0.48	0.31 (0.14, 0.71)	0.005
2:110177146	rs1154652	*NPHP1*	Intronic	G	0.35	0.14	3.41 (1.46, 7.96)	0.005
9:69254557	rs3812536	*TJP2*	UTR3	A	0.68	0.42	3.01 (1.36, 6.66)	0.007
12:100559789	rs35728	*NR1H4*	Intronic	A	0.63	0.37	2.89 (1.34, 6.25)	0.008

Abbreviations: AF, allele frequency; Chr:BP, chromosome and basepair; OR (95% CI), odds ratio (95% confidence interval); SNP, single nucleotide polymorphism.

## 4 Discussion

Hormonal contraceptives are a diverse group that extends beyond just the combined oral contraceptives used for birth control. After the initial report from the Women's Health Initiative showed more harmful than beneficial effects, hormone replacement therapy fell out of favour. Currently, oestrogen monotherapy is mainly used for menstrual disorders, especially postmenopausal bleeding. In this report, we focus primarily on hormonal contraceptives used for women's health, such as birth control and postmenopausal bleeding. Even within this narrower scope, we found that hormonal agents differed greatly in their hormonal composition (single versus combined) and modes of delivery (oral, transdermal, or subdermal implant). Additionally, the daily oestrogen dosage varied in cases involving single or combination drug regimens. Interestingly, from the perspective of DILI, there is also variation in hormone doses administered throughout the cycle. For example, a monophasic hormonal agent provides a fixed dose of oestrogen and progestin, whereas a triphasic hormonal agent has three hormone dose levels that change across cycle phases.

DILI from hormonal agents was uncommon, affecting about 1% of the current cohort. It can occur with oestrogens alone, progesterone‐only, or combination contraceptives, using various delivery methods and dosages. Several important themes emerge from this study. The route of administration does not eliminate the risk, as transdermal contraceptive patches, often regarded as safer, can still cause significant liver injury. Additionally, cases where intravenous hormonal therapy followed by oral contraceptive use demonstrated prolonged or even chronic cholestasis. Other key clinical observations related to DILI risk in this cohort include: (a) low‐dose oestrogen‐containing OCs primarily showing a hepatocellular or mixed injury pattern, (b) no link with phasic delivery of OCs, (c) chronic progressive cholestatic DILI from oestrogen monotherapy, and (d) potential cumulative toxicity from concurrent use of oestrogens via oral, transdermal or subdermal implants. Compared to matched controls, the hormonal agents group showed a distinct mixed or cholestatic pattern, with patients more often presenting symptoms such as jaundice and pruritus. Systemic inflammatory signs, like fever, were notably absent, and reports of rash and elevated autoimmune markers were less common. The median onset of liver injury was 36 days, occurred earlier than with the control arm and the cohort reported by Patil M et al. [6].

In this analysis, variants enriched in cases were mapped to *MROH2A* (Maestro Heat‐Like Repeat Family Member 2A), a poorly characterised gene that might influence nearby liver‐expressed genes through regulatory effects. Variants also mapped to *NPHP1* (Nephrocystin‐1), a ciliopathy gene expressed in cholangiocytes that can affect ductal signalling and bile flow, *TJP2* (Tight Junction Protein 2/ZO‐2), a protein that maintains canalicular tight junctions and helps prevent paracellular bile leaks, and *NR1H4* (Nuclear Receptor Subfamily 1 Group H Member 4; farnesoid X receptor, FXR), the primary bile acid receptor that activates transporters like *BSEP* (bile salt export protein) and reduces bile acid synthesis. Conversely, alleles depleted in cases (possibly protective) were found in *AKR1D1* (Aldo‐Keto Reductase Family 1 Member D1; steroid 5β‐reductase), a key enzyme in bile acid production that shapes the bile acid pool, and *ABCG8* (ATP‐Binding Cassette Subfamily G Member 8), which collaborates with *ABCG5* to export sterols into bile and affect bile composition. Overall, these loci influence bile acid homeostasis, canalicular barrier function, and cholangiocyte biology, providing a plausible biological basis for oestrogen‐triggered cholestasis, while highlighting that the signals are likely regulatory (intronic/3′UTR) and need replication and functional validation.

DILI from hormonal agents seems to share a common clinical pattern of hormone‐triggered cholestasis, like intrahepatic cholestasis of pregnancy (ICP). Biochemically, both conditions predominantly exhibit a cholestatic pattern of liver injury [15]. Pruritus is a prominent symptom in both conditions and can appear before jaundice [15]. Both disorders tend to recur with re‐exposure: future pregnancies in ICP or reintroduction of hormonal agents [15]. This similarity suggests an underlying host predisposition. Key differences include timing and outcomes: ICP usually occurs in the late second or third trimester and resolves quickly after delivery, while hormonal contraceptive DILI may happen weeks or months after exposure [15]. In ICP, serum bile acids are central to diagnosis and risk stratification, while ALP is less informative due to physiologic elevation during pregnancy; GGT is often normal or only mildly elevated [15]. In contrast, the hormonal agent‐associated liver injury more consistently shows elevations in ALP and GGT, with bile acids measured less routinely. Hormonal agent‐associated liver injury also exhibits greater heterogeneity, ranging from pure cholestasis to mixed or even hepatocellular patterns, depending on the hormonal formulation and individual susceptibility.

Genetically, both conditions are enriched for variants affecting bile acid transport and canalicular function, particularly in genes such as *ABCB11*, *ABCB4* and *ATP8B1* [16, 17]. These shared pathways support the idea of hormonally unmasked cholestatic predisposition. ICP often reflects a polygenic risk that becomes clinically evident under the high hormonal burden of pregnancy, whereas hormonal agent‐associated DILI appears more exposure‐dependent, with penetrance influenced by hormone dose, formulation and route of administration [16, 17]. A history of ICP may serve as an important clinical indicator for increased susceptibility to cholestasis when using exogenous hormonal therapies.

The current report did not include hormonal agents for indications beyond women's health. Additionally, our report excluded agonists such as selective oestrogen receptor modulators (SERMs) and selective progesterone receptor modulators (SPRMs), which have oestrogenic effects on bone and serum lipids, neutral effects on the uterus and anti‐oestrogenic effects on breast tissue [18]. However, these agents are not classified as hormonal agents. The history of pregnancy‐related liver disease was recorded in two cases, both from the same centre, with one case undergoing genetic testing. Unfortunately, pregnancy‐related liver disease was not documented in all cases, and for some, it was their first pregnancy. Unlike the mortality reported by Patel M et al. from India, we did not observe any mortality associated with the use of these agents [6]. In contrast to the Indian registry, we did not identify any cases associated with ormeloxifene, likely because it is marketed exclusively in India. These differences in implicated agents likely reflect regional variations in drug availability and prescribing practices [6].

In summary, we confirm previous findings of a latency period of 2–3 months and resolution of injury around 2 months after stopping the suspected agent. The current report and selected case summaries show that all three patterns of liver enzyme elevations in DILI are associated with hormonal contraceptives used through various methods, including transdermal patches. A comparison with the control group allowed us to identify a clinical presentation less likely to be associated with systemic inflammatory symptoms and more likely to present with jaundice and require hospitalization. The link to genes involved in bile acid regulation, canalicular tight junctions, and cholangiocyte function provides a biological model for hormone contraceptive‐associated cholestatic DILI; however, the regulatory signals (intronic/3′UTR) need to be replicated and functionally validated as the current study was not adequately powered for it.

## Author Contributions

Raj Vuppalanchi and Naga Chalasani conceived and designed the study. Raj Vuppalanchi and A. Sidney Barritt contributed to the acquisition of clinical data. Huiman Barnhardt, Sherry Gu, and Yi‐Ju Li performed the statistical analysis and data management. Raj Vuppalanchi drafted the manuscript. A. Sidney Barritt, Anjali Rai, Huiman Barnhardt, Sherry Gu, Yi‐Ju Li, and Naga Chalasani critically revised the manuscript for important intellectual content. Naga Chalasani supervised the study. All authors approved the final version of the manuscript for submission and agree to be accountable for all aspects of the work.

## Funding

Research reported in this publication was supported by the National Institute of Diabetes and Digestive and Kidney Diseases (NIDDK) of the National Institutes of Health (NIH) under award numbers U01DK065211 (Indiana University), U01DK065184 (University of Michigan), U01DK065201 (University of North Carolina–Chapel Hill), U01DK083020 (University of Southern California), U01DK083027 (Thomas Jefferson University/Albert Einstein Medical Center), and U24DK065176 (Duke University). The Drug‐Induced Liver Injury Network (DILIN) is structured as a U01 cooperative agreement, with additional support provided by the NIDDK intramural programs and the National Cancer Institute. The participating institutions include Indiana University [Purdue], the University of Michigan [Ann Arbor], the University of North Carolina [Chapel Hill], the University of Southern California, Thomas Jefferson Health/Albert Einstein Medical Center, and Duke Clinical Research Institute. The content is solely the authors' responsibility and does not necessarily represent the official views of the National Institutes of Health.

## Conflicts of Interest

Naga Chalasani reports no conflicts of interest related to this paper. For full disclosure, he reports consulting agreements with Madrigal, GSK, Ipsen, Zydus, Eccogene, Insitro, Biomea Fusion, and Chugai. He reports research support from Madrigal, Boehringer Ingelheim, and Exact Sciences. He has equity interests in Avant Sante, a contract research organization, and Heligenics, a drug‐discovery start‐up. Raj Vuppalanchi reports no conflicts of interest related to this paper. However, for full disclosure, he receives institutional research funding from Eli Lilly, Galectin Therapeutics, Takeda, Zydus Therapeutics Inc., and GlaxoSmithKline. He also serves as a consultant for Fortrea, Medpace, Thermo Fisher, Madrigal Pharmaceuticals, Ipsen, Akero Therapeutics, Regeneron, 89 Bio, GSK, Cogent Biosciences, Agios, and Takeda in various roles, including on steering committees, data safety monitoring committees, advisory boards, and adjudication committees related to hepatic safety and clinical outcomes.

## Supporting information


**Table S1:** Cholestasis gene panel: genes and full names. Common protein aliases are shown in parentheses. This table summarizes genes frequently evaluated in cholestatic phenotypes.

## Data Availability

Data are available upon request from the Central Repository of NIDDK (https://repository.niddk.nih.gov/home).
