# Supplementary material for: Liver Injury Due to Hormonal Contraceptives in the Modern Era
Source: Liver Int. 2026 Nov 2;46(12):e70945. doi: 10.1111/liv.70945 (PMC13726578; doi:10.1111/liv.70945)
Supplement: Supplementary file 1 [file LIV-46-0-s001.docx]

**Supplementary Table 1.** Cholestasis Gene Panel: Genes and Full Names. Common protein aliases are shown in parentheses. This table summarizes genes frequently evaluated in cholestatic phenotypes.

| **Gene** | **Full name** |
| --- | --- |
| ABCB11 | ATP-binding cassette subfamily B member 11 (BSEP) |
| ABCB4 | ATP-binding cassette subfamily B member 4 (MDR3) |
| ABCC2 | ATP-binding cassette subfamily C member 2 (MRP2) |
| ABCG5 | ATP-binding cassette subfamily G member 5 |
| ABCG8 | ATP-binding cassette subfamily G member 8 |
| ACOX2 | Acyl-CoA oxidase 2, peroxisomal (branched-chain) |
| AKR1D1 | Aldo-keto reductase family 1 member D1 (steroid 5β-reductase) |
| ALDOB | Aldolase, fructose-bisphosphate B |
| AMACR | Alpha-methylacyl-CoA racemase |
| ATP8B1 | ATPase phospholipid transporting 8B1 (FIC1) |
| BAAT | Bile acid-CoA:amino acid N-acyltransferase |
| CC2D2A | Coiled-coil and C2 domain containing 2A |
| CFTR | Cystic fibrosis transmembrane conductance regulator |
| CLDN1 | Claudin 1 |
| CYP27A1 | Cytochrome P450 family 27 subfamily A member 1 |
| CYP7B1 | Cytochrome P450 family 7 subfamily B member 1 |
| DCDC2 | Doublecortin domain containing 2 |
| DGUOK | Deoxyguanosine kinase |
| DHCR7 | 7-Dehydrocholesterol reductase |
| FAH | Fumarylacetoacetate hydrolase |
| GNAS | GNAS complex locus (G protein subunit alpha s) |
| HNF1B | HNF1 homeobox B |
| HSD17B4 | Hydroxysteroid 17-beta dehydrogenase 4 |
| HSD3B7 | 3-Beta-hydroxysteroid dehydrogenase type 7 |
| JAG1 | Jagged canonical Notch ligand 1 |
| KMT2D | Lysine methyltransferase 2D (MLL2) |
| LIPA | Lipase A, lysosomal acid lipase |
| MKS1 | Meckel syndrome type 1 protein |
| MPV17 | MPV17 mitochondrial inner membrane protein |
| MYO5B | Myosin VB |
| NOTCH2 | Notch receptor 2 |
| NPC1 | Niemann-Pick disease, type C1 |
| NPC2 | Niemann-Pick disease, type C2 |
| NPHP1 | Nephrocystin-1 |
| NPHP3 | Nephrocystin-3 |
| NPHP4 | Nephrocystin-4 |
| NR1H4 | Nuclear receptor subfamily 1 group H member 4 (FXR) |
| PEX1 | Peroxisomal biogenesis factor 1 |
| PEX2 | Peroxisomal biogenesis factor 2 |
| PEX3 | Peroxisomal biogenesis factor 3 |
| PEX5 | Peroxisomal biogenesis factor 5 |
| PEX6 | Peroxisomal biogenesis factor 6 |
| PEX7 | Peroxisomal biogenesis factor 7 |
| PEX10 | Peroxisomal biogenesis factor 10 |
| PEX11B | Peroxisomal biogenesis factor 11 beta |
| PEX12 | Peroxisomal biogenesis factor 12 |
| PEX13 | Peroxisomal biogenesis factor 13 |
| PEX14 | Peroxisomal biogenesis factor 14 |
| PEX16 | Peroxisomal biogenesis factor 16 |
| PEX19 | Peroxisomal biogenesis factor 19 |
| PEX26 | Peroxisomal biogenesis factor 26 |
| PKD1L1 | Polycystin 1 like 1 |
| PKHD1 | Polycystic kidney and hepatic disease 1 (fibrocystin) |
| POLG | DNA polymerase gamma, catalytic subunit |
| SCYL1 | SCY1 like pseudokinase 1 |
| SERPINA1 | Serpin family A member 1 (alpha-1 antitrypsin) |
| SLC10A1 | Solute carrier family 10 member 1 (NTCP) |
| SLC10A2 | Solute carrier family 10 member 2 (ASBT/IBAT) |
| SLC25A13 | Solute carrier family 25 member 13 (citrin) |
| SLC27A5 | Solute carrier family 27 member 5 (FATP5) |
| SLC51B | Solute carrier family 51 beta subunit (OSTβ) |
| SLCO1B3 | Solute carrier organic anion transporter family member 1B3 (OATP1B3) |
| SMPD1 | Sphingomyelin phosphodiesterase 1 |
| TALDO1 | Transaldolase 1 |
| TJP2 | Tight junction protein 2 (ZO-2) |
| TMEM216 | Transmembrane protein 216 |
| TRMU | tRNA 5-methylaminomethyl-2-thiouridylate methyltransferase |
| UGT1A1 | UDP-glucuronosyltransferase family 1 member A1 |
| VIPAS39 | VPS33B interacting protein, apical-basolateral polarity regulator (SPE-39) |
| VPS33B | VPS33B, CORVET/HOPS core subunit |

**Abbreviations:** BSEP, bile salt export pump; MDR3, multidrug resistance protein 3; MRP2, multidrug resistance-associated protein 2; FIC1, familial intrahepatic cholestasis protein 1; NTCP, sodium taurocholate co-transporting polypeptide; ASBT/IBAT, apical sodium-dependent bile acid transporter/ileal bile acid transporter; FATP5, fatty acid transport protein 5; OSTβ, organic solute transporter beta; OATP1B3, organic anion transporting polypeptide 1B3.

**Note:** Aliases in parentheses are provided for clinical familiarity and do not imply standardized nomenclature across all laboratories.

**Reference**

https://www.preventiongenetics.com/tests/cholestasis-panel?tab=genes-%26-conditions
